# Myocardial Calcifications: An Exceptional Complication of Sepsis

**DOI:** 10.7759/cureus.49977

**Published:** 2023-12-05

**Authors:** Zakia El Yousfi, Hamza Chraibi, Omar El Aoufir, Laila Jroundi, Fatima Zahrae Laamrani

**Affiliations:** 1 Radiology, Ibn Sina University Hospital Center, Rabat, MAR; 2 Cardiology, Mohammed V University, Rabat, MAR

**Keywords:** transthoracic echocardiography (tte), ct finding, imaging, sepsis, myocardial calcifications

## Abstract

Myocardial calcifications (MCs) are a fatal condition that often complicates ischemic heart disease, cardiac surgery, rheumatic fever, or myocarditis. To date, cases where myocardial calcifications result from a septic state have rarely been reported. In this paper, we describe the primary imaging findings and discuss both proven and hypothetical mechanisms of MCs in the context of sepsis.

## Introduction

Myocardial calcifications (MCs) constitute a life-threatening condition, commonly arising as complications of ischemic heart disease, cardiac surgery, rheumatic fever, or myocarditis. It is important to note that MCs differ from coronary artery or valve calcifications and are observed in patients with abnormalities in calcium metabolism [1]. While their occurrence in the context of sepsis is rare, etiopathogenesis remains poorly understood [2].

In this paper, we present a case involving a patient who developed MCs subsequent to postoperative sepsis and provide an in-depth discussion on the computed tomography (CT) aspects of the case.

## Case presentation

A 25-year-old man presented with abdominal pain that had been progressing over the past five days before his admission, accompanied by episodes of vomiting. He was admitted to the emergency department for further exploration. The initial physical examination revealed stable vital signs, a generalized abdominal contracture, and diffuse tenderness.

**Figure 1:**
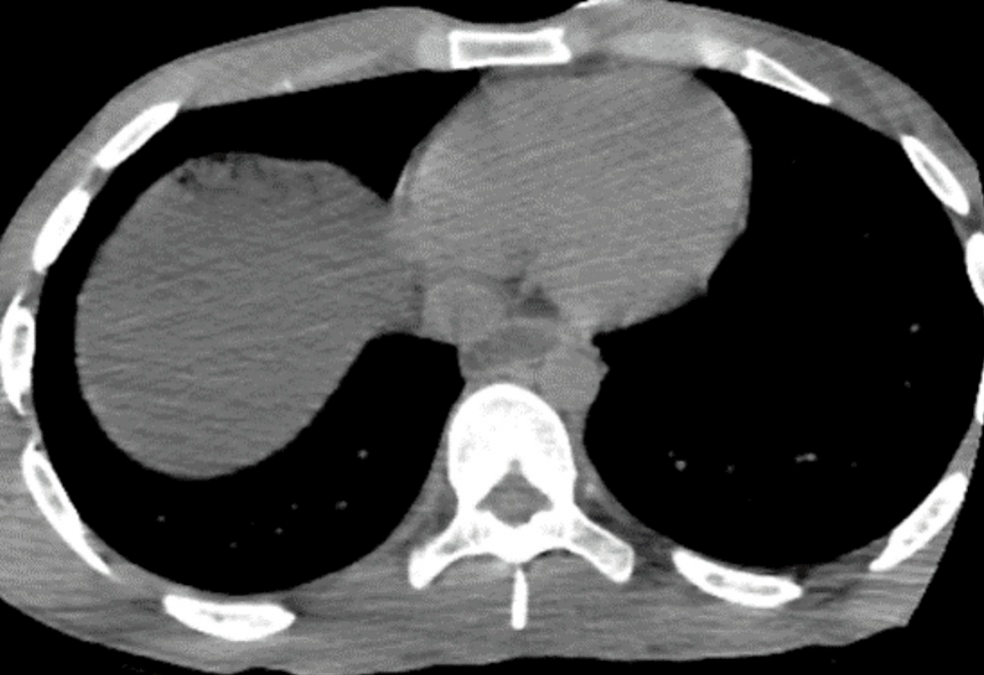
Axial noncontrast-enhanced CT of the thorax on day 1 showing no abnormalities.

**Figure 2:**
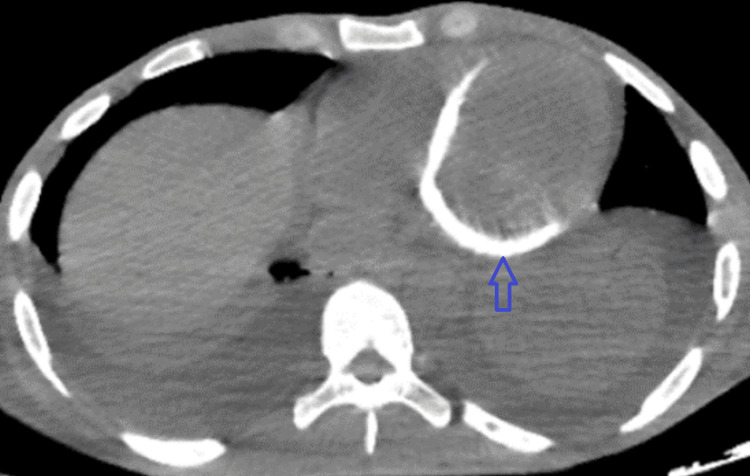
Noncontrast-enhanced CT scan of the chest on day 29 with the axial view showing extensive myocardial calcifications of the left ventricular wall associated with bilateral pulmonary pleural effusion. Blue arrow: myocardial calcifications of the left ventricle

An abdominal CT scan indicated a large intraperitoneal effusion with pneumoperitoneum, confirming the diagnosis of acute generalized peritonitis. A chest CT scan showed no abnormalities (Figure 1). The patient underwent emergent laparotomy, revealing a perforation of the duodenal bulb. In the early postoperative period, the patient developed septic shock and multiple organ failure. Treatment in the intensive care unit involved broad-spectrum antibiotics and vasopressors. Two weeks later, the patient fully recovered and was transferred to the general surgery department, where a control CT scan revealed diffuse intramyocardial calcifications in the left ventricular walls, without coronary distribution, and bilateral pleural effusion (Figure 2). Physical examination and electrocardiogram were normal. Transthoracic echocardiography (TTE) identified global left ventricular hypokinesia with an estimated ejection fraction of 40%, without significant valvular disease. Biological findings, including inflammatory markers and the phosphocalcic panel, were normal. The patient was discharged after an uneventful hospital course, and no therapeutic interventions were proposed at the time.

One month after discharge, the patient attended an early cardiology follow-up. Physical examination was normal, with no signs of heart failure (HF). TTE showed no improvement in left ventricular function. Conventional HF therapy, consisting of ramipril, bisoprolol, spironolactone, and dapagliflozine, was initiated.

## Discussion

MCs of septic origin constitute a rare condition, and their etiology remains incompletely explained. To date, two recognized pathogenic mechanisms are dystrophic and metastatic calcifications [3].

Dystrophic calcifications arise as a consequence of myocardial pathology (necrosis or fibrosis) and have been documented following myocardial infarction, surgery, trauma, and myocarditis. They may also manifest in patients who have survived severe sepsis, as observed in our reported case.

Metastatic calcifications, on the other hand, result from abnormalities in serum calcium and can lead to calcium deposits in various organs such as the skin, lungs, stomach, and kidneys [4]. Notably, our patient lacked laboratory evidence indicating a significant disturbance in calcium-phosphate balance, and the calcium deposits were confined to a single organ, the myocardium.

The proposed mechanism suggests that sepsis induces microcirculation disturbance and cell lysis, with inflammation and infiltration of macrophages and giant cells synergistically contributing to calcification production [5]. Other studies have indicated that septic shock releases toxins and cytokines, causing alterations in cell membranes, leading to calcium influx into cardiomyocytes, and resulting in mitochondrial dysfunction and myocardial necrosis [6]. Additionally, the potential side effects of amines injected into patients cannot be ruled out, as they may also damage cardiomyocyte membranes [7].

Chest CT scans play a crucial role in diagnosing MCs in the context of sepsis. This modality is highly recommended when conventional chest radiographs or echocardiography yield uncertain findings due to its superior contrast resolution and lack of projections of superimposed structures on axial planes [6]. In our case, MCs were incidentally discovered on thoracic imaging, appearing as extensive linear left ventricular MCs. Dystrophic calcifications were noted to be linear, focal, and localized to the organ, in contrast to the more diffuse and unstructured nature of metastatic calcifications.

Infections and inflammatory etiologies often present as linear or diffuse circumferential deposits, consistent with our case. MCs are frequently described in the outer layers of the left ventricle (apex, septum, anterolateral, and inferior walls) and rarely in the right cavities. TTE may reveal calcifications of the ventricular walls or indicate left ventricular systolic dysfunction [8].

As of now, there are no clearly defined therapies for the prevention or treatment of this cardiac complication. Guideline-directed medical therapy for HF should be prescribed in the presence of left ventricular dysfunction or congestive symptoms, although there is no specific data for this subset of patients. Pericardiectomy may be needed in case of constrictive pericarditis. The prognosis of our patient remains poor, as with all other cases of HF with reduced ejection fraction [9].

## Conclusions

MCs are a rare manifestation in the setting of severe systemic sepsis, and the mechanisms responsible for their development are poorly understood. Signs of this complication should be diligently detected in patients who have survived the acute phase of severe sepsis through careful monitoring with imaging modalities such as CT.
